# Primary Ovarian Leiomyoma in a White Tiger (*Panthera tigris*)

**DOI:** 10.3390/vetsci9120702

**Published:** 2022-12-17

**Authors:** Carmelo Iaria, Antonio Ieni, Luca Cicero, Giovanni Briguglio, Salvatore Di Maria, Jessica Maria Abbate

**Affiliations:** 1 Department of Chemical, Biological, Pharmaceutical and Environmental Sciences, University of Messina, Polo Universitario Papardo, 98166 Messina, Italy; 2 Department of Human Pathology of Adult and Evolutive Age “Gaetano Barresi”, Section of Pathology, University of Messina, 98125 Messina, Italy; 3 Zooprophylactic Institute of Sicily “A. Mirri” (IZS), 90129 Palermo, Italy; 4 Department of Veterinary Sciences, University of Messina, Polo Universitario Annunziata, 98168 Messina, Italy; 5 Veterinary Practitioner, 96100 Syracuse, Italy

**Keywords:** leiomyoma, ovarian leiomyoma, ovary, tumours, white tiger, *Panthera tigris*, non-domestic felids, contraceptives, GnRH analogues

## Abstract

**Simple Summary:**

Reproductive system cancers occur frequently in captive non-domestic felids, negatively impacting wildlife conservation by affecting reproduction. Ovarian tumours are very rare and occasionally mentioned in retrospective studies of large numbers of wild felids aimed at determining tumour types, prevalence rates, and species distribution. In this report, we describe the occurrence of an ovarian leiomyoma in a 22-year-old white tiger (*Panthera tiger*) treated with a gonadotropin-releasing hormone (GnRH) agonist to control reproduction, detailing histomorphological and immunohistochemical characteristics. Reproductive cancer affects reproduction, and since maintenance of fertility in large felids is essential for captive breeding programs, it is important to regularly monitor animals for development of neoplasia, allowing for early diagnosis and effective management. Finally, the possible role of contraceptives in the pathogenesis of cancer in non-domestic felids is worthy of future investigation.

**Abstract:**

Ovarian leiomyomas are very rare in domestic cats and occasionally mentioned in studies reporting general pathological findings and neoplasm occurrence in non-domestic large felids. This report describes a case of ovarian leiomyoma in a 22-year-old white tiger (*Panthera tiger*), treated with deslorelin implants, detailing pathological and immunohistochemical characteristics. Gross examination revealed a markedly enlarged, firm, whitish right ovary with a multinodular appearance. On a cut surface, multiple brown-fluid-filled cysts interspersed with solid grey-to-white areas were observed. On histopathological examination, the ovary was enlarged and replaced by a densely cellular neoplasm composed of spindle cells arranged in fascicles, or occasionally in a herringbone pattern, embedded in a large stroma of collagenous connective tissue. Neoplastic cells showed mild nuclear atypia and pleomorphism and low mitotic rate. Immunohistochemistry confirmed smooth muscle origin of the neoplasm, and cells were positive for vimentin, alpha-smooth muscle actin, desmin, and caldesmon. A low rate (<1%) of Ki-67-positive cells was observed. Although rare, ovarian leiomyoma should be considered when a mass is present in the ovary of a tiger with reproductive failure. Because cancer of the reproductive system impacts on species conservation by affecting reproduction, regular health monitoring is warranted to support wildlife conservation. Finally, the adverse effects associated with long-term treatment with synthetic GnRH analogues as contraceptives in non-domestic felids are worthy of future investigation.

## 1. Introduction

Neoplastic disease causes high morbidity and mortality rates in wildlife [1], and the incidence of cancer appears to be higher in captive animals than in their wild counterparts due to their longer life expectancy resulting from improved veterinary management and care [2]. Currently, an ever-increasing number of wild animals live in zoos and zoological gardens located in urbanized areas, and the exposure to various environmental factors, such as chemical carcinogens, contaminants, and radiation, may influence the development of cancer in wildlife as well [3,4]. Furthermore, genetic aberrations, infectious agents, and the widespread use of contraceptives to control reproduction are causes of an increase in the rate of tumours in captive wild species [1,5,6,7].

In non-domestic felids, neoplasms are common and often malignant [8,9]. A great diversity of cancers has been described in large felids and tumours of the reproductive system are among the most observed, with a higher risk in older animals, suggesting that age has a major impact on tumour occurrence [8,9,10,11]. The prevalence of cancer affecting the reproductive system is particularly highlighted because diseases of the reproductive organs can negatively impact species conservation by affecting reproduction.

Particularly, in tiger (*Panthera tigris*), mammary tumours account for almost 60% of all reproductive neoplasms, followed by uterine neoplasms with a high prevalence of leiomyomas [10,11]. Leiomyomas are benign tumours consisting of smooth muscle cells with a varying amount of fibrous tissue. In the reproductive system, leiomyomas arising from the uterus are frequently diagnosed in domestic animals and noteworthy uterine leiomyomas are commonly detected in older tigers during autopsy [8,9,10,11]. In contrast, primary leiomyomas of the ovary are rare and occasionally mentioned in previous studies listing general pathological findings and neoplasm occurrence in large felids, including tigers, with the aim of determining tumour types, prevalence rates, and species distribution [8,9,10,11,12]. Since ovarian leiomyoma has a very low prevalence, its pathological features have rarely been described in captive wildlife [12]. Therefore, this report describes the pathological and immunohistochemical characteristics of a case of ovarian leiomyoma in a captive white tiger (*Panthera tigris*).

## 2. Case Presentation

A 22-year-old, intact female white tiger (*Panthera tigris*) kept in a circus in the province of Catania (Sicily, Italy), was presented to the Department of Veterinary Sciences of the University of Messina for post-mortem investigations. The owner reported that the tiger suffered from severe recurrent respiratory symptoms in the last three years of its life, and showed nonspecific clinical signs in the previous two weeks, including anorexia, weight loss, and weakness with permanent lateral recumbency. The tiger was euthanized for poor health conditions. The tiger received regular vaccinations for *Felid Herpesvirus* 1, *Feline calicivirus,* and *Feline panleukopenia* until the age of 4, was periodically treated to control internal parasites, and was treated for up to 4 years with deslorelin implants (Suprelorin^®^, Virbac) for reproduction control.

At the autopsy, a general examination found the tiger to be in poor body condition, with depletion of subcutaneous and visceral fat stores and hypotonic skeletal muscle mass. Body weight was estimated at 160 kg. Diffusely, the coat was dry and brittle with a dusty appearance, and multiple ulcerative lesions were observed on the skin covering the bony prominences on the left side—including the scapular region, hemithorax, and left hind limb—representing compression-induced skin lesions (pressure ulcers) due to prolonged lateral decubitus. All mucous membranes were pale pink/white on physical examination.

Upon opening the abdominal cavity along the midline, the right ovary was 23 cm × 18 cm × 6 cm in size, partially firm, whitish, with a multinodular appearance, and characterized by extensive peritoneal adhesions with adjacent abdominal viscera (i.e., liver; intestine). The cut surface had multiple cystic cavities, varying in size, and filled with yellow to brownish fluid interspersed between firm grey/white to mottled dark red solid areas. A diffuse and moderate dilation of the uterus was noted, with a thinner and paler uterine wall, and accumulation of viscid fluid inside, compatible with mucometra. No macroscopic metastases were found in other organs. Additional findings during gross pathological examination included: chronic, diffuse, and severe bronchopneumonia with multifocal areas of alveolar emphysema and oedema and severe cardiac dilation.

Representative portions of the ovarian mass were sampled and fixed in 10% neutral buffered formalin, and after trimming, samples were routinely embedded in paraffin for histopathological examination. Paraffin sections 3 μm thick were stained with hematoxylin and eosin (HE) and Masson’s Trichrome stain using a commercially available kit (DIAPATH; Bergamo, Italy) and following manufacturer’ instructions.

**Figure 1:**
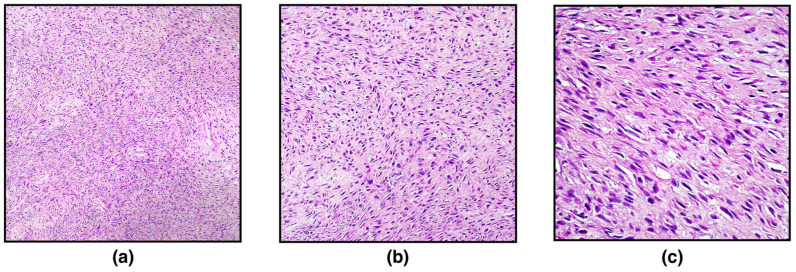
Leiomyoma, ovary, white tiger. Hematoxylin and eosin (HE). (**a**) Neoplastic cells are arranged in interlacing fascicles and in a herringbone pattern, embedded in a collagenous connective tissue (magnification, 100×); (**b**) spindle-shaped neoplastic cells showed minimal atypia and polymorphism (magnification, 200×); (**c**) higher magnification (400×).

On histopathological examination, the ovary was markedly expanded and replaced by a well-demarcated, non-encapsulated, moderately cellular neoplasm composed of densely packed spindle-shaped neoplastic cells arranged in interlacing fascicles or showing a herringbone pattern and embedded in a large stroma of collagenous connective tissue. Spindle cells were 10–15 μm long with indistinct cytoplasmic borders, scant eosinophilic cytoplasm, and eccentric, elongated, “cigar-shaped” nuclei with finely stippled chromatic and occasionally distinct nucleoli. Neoplastic cells showed mild nuclear atypia and cellular pleomorphism. Mitotic figures were 2 in 10 high-power fields (400×; 2.37 mm^2^) (Figure 1). Scattered, small, multifocal haemorrhages and multifocal infiltration of scant lymphocytes, plasma cells, and macrophages have been observed. Based on the histopathological characteristics, a diagnosis of ovarian leiomyoma was made, further confirmed with Masson’s Trichrome staining and immunohistochemistry.

**Figure 2:**
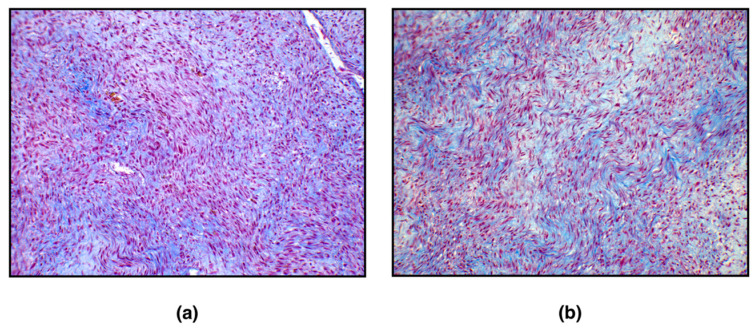
Leiomyoma, ovary, white tiger. Masson’s Trichrome (MT). (**a**) Smooth muscle cell cytoplasm was stained red (magnification, 200×); (**b**) abundant, thin, blue-stained collagen bundles intersect between individual neoplastic cells (magnification, 200×).

With the Masson Trichrome staining, the cytoplasm of smooth muscle cells was stained red, and thin, abundant, blue-stained collagen bundles intersected between individual neoplastic cells (Figure 2).

Immunohistochemistry (IHC) was performed on paraffin-embedded 3 μm thick tissue sections, using the Ventana BenchMark ULTRA automated platform with cell conditioning 1 for 64 min and pre-peroxidase inhibition and primary antibody incubation for 16 min at 37 °C. The OptiView DAB IHC Detection kit (Ventana Medical Systems, Inc.) was used to detect protein expression of the following primary antibodies: vimentin (clone V9, catalogue number 790–2917); alpha-smooth muscle actin (clone 1A4, catalogue number 760–2833); desmin (clone DE–R–11 catalogue number 760–2513); caldesmon (clone E89, catalogue number 760–4375); Ki67 (clone 30–9, catalogue number 790–4286); inhibin, alpha (clone MRQ–63, catalogue number 760–6081). Slides were counterstained with Haematoxylin II (Ventana Medical Systems, Inc.) and Bluing Reagent (Ventana Medical Systems, Inc.) for 4 min at room temperature. Negative controls were obtained by omitting the specific antisera and substituting PBS for the primary antibody. Suitable positive controls were used for the IHC reactions.

**Figure 3:**
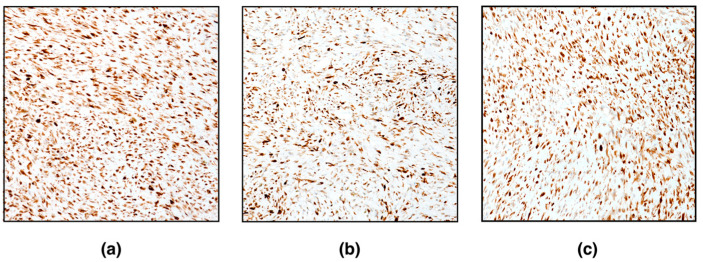
Immunohistochemical characterization of leiomyoma, ovary, white tiger. (**a**) Vimentin, (**b**) desmin, and (**c**) caldesmon expression (magnification 200×).

Immunohistochemically, spindle neoplastic cells were positive for vimentin, alpha-smooth muscle actin, desmin, and caldesmon (Figure 3). Conversely, neoplastic cells were negative for inhibin-alpha. Very few (< 1%) proliferating neoplastic cells (Ki67-positive cells) were detected. Based on histomorphological features and immunohistochemistry, the diagnosis of ovarian leiomyoma was confirmed.

## 3. Discussion

Ovarian cancers have been described in all domestic species, although they are rare, and this most likely could reflect the high proportion of spayed animals examined by veterinarians rather than an inherent resistance of the ovaries to tumour development [13]. The classification of primary ovarian tumours in domestic animals is based on the embryological derivation of the cells and includes: sex cord stromal tumours (e.g., granulosa cell tumour, luteoma, thecoma), germ cell tumours (e.g., dysgerminoma; teratoma), and epithelial tumours arising from the lining ovarian epithelium, rete ovarii and subsurface epithelial structures (SES) [14]. Tumours arising from supporting tissue, including fibroblastic, smooth muscle, and vascular tumours are rare, except for ovarian hemangiomas in older sows [15,16]. Finally, metastatic tumours, particularly lymphomas, can secondarily invade the ovary [17]. Typically present in older animals, ovarian neoplasms can be asymptomatic, representing an accidental finding during various diagnostic investigations and/or autopsies, or—if endocrinologically functional (e.g., granulosa cell tumour)—tumours may be associated with behavioural changes or changes in target tissues [14].

Here, we detail pathological and immunohistochemical features of an ovarian leiomyoma in a white tiger (*Panthera tigris*) as an accidental finding at necropsy. Previous retrospective studies reporting general pathological findings and neoplasms in a cohort of large felids have rarely and summarily reported ovarian leiomyoma in *Panthera* species, such as lion (*Panthera leo*), leopard (*Panthera pardus*), and tiger (*Panthera* tigris) [8,10,11,12]. In contrast, leiomyoma is the most common mesenchymal tumour in the uterus in *Panthera* species, representing a common gross pathological finding at autopsy [8,9,10,11]. Reproductive tract leiomyomas are commonly found in older animals (median age 17 years), suggesting that increasing age likely had a major impact on tumour prevalence, and they usually predispose to secondary complications such as hydrometra, mucometra and pyometra, resulting in reproductive failure [8,9,10,11].

Morphological features that support the diagnosis of ovarian leiomyoma include the arrangement of cells in intertwined bundles, occurring in both long and cross sections, with occasional herringbone pattern of growth. Neoplastic cells resembled smooth muscle with scant eosinophilic cytoplasm and “cigar-shaped” nuclei with blunt ends. The main differential diagnoses for the tumour described here included well-differentiated leiomyosarcoma, thecoma, and fibroma. Although it may be composed of spindle-shaped cells that maintain the morphology of normal smooth muscle cells with mild pleomorphism, well-differentiated leiomyosarcoma is generally more cellular, and mitoses can be conspicuous (1–2 in high power field) [14]. In addition to mitotic rate, the evidence for vascular or lymphatic invasion and tumour cell necrosis are the main histological features useful in distinguishing well-differentiated leiomyosarcoma from leiomyoma [14]. The distinction between thecoma and mesenchymal tumours, such as ovarian leiomyoma and fibroma, can be difficult on HE-stained slides, and special stains and IHC are useful for identifying neoplastic cells [14]. The absence of IHC labelling for inhibin alpha found here, was useful to distinguish mesenchymal tumours from gonadal sex cord-stromal tumours (i.e., thecoma). Smooth muscle differentiation of cells was confirmed via IHC, which found cells strongly positive for alpha-smooth muscle actin and caldesmon, an actin-binding protein considered a specific marker for tumours with smooth muscle differentiation [18]. The use of caldesmon has been validated in diagnosis of genital smooth muscle tumours in women and recently also explored in domestic cat [19,20].

In large felids, a causative role of hormonal treatments in cancer of the reproductive system has been suggested, although data in the literature are sometimes conflicting [6,7,8,12,21]. In particular, progestin-based contraceptives are largely administered in large felids for the reproductive control of genetically valuable animals, and, in particular, it has been suggested that melengestrol acetate (MGA) is responsible for the high incidence of malignant tumours of the reproductive system [21]. It is noteworthy that 94% of the captive wild felids with mammary cancer had received MGA [22], as well as MGA administration was associated with uterine carcinomas and leiomyosarcomas in zoo felids [6,7].

The tiger included in this study was treated with a GnRH agonist (deslorelin acetate; implants), which acts by suppressing ovarian follicular activity for 4–14 months [23]. In general, deslorelin acetate has been showed to be effective and safe in domestic cat and captive-held wild felids, although permanent infertility may occur [23,24,25,26]. Conversely, the occurrence of ovarian tumours and uterine diseases (*e.g.,* mucometra, pyometra) have been associated with long-term treatment with deslorelin in bitches [27]. Therefore, the role of synthetic GnRH agonists, such as deslorelin acetate in cancer pathogenesis in non-domestic felids is worthy of future investigations, and surveillance for immediate as well as long-term contraceptive-associated adverse effects should be conducted regularly.

Ovariohysterectomy is the method of choice for preventing the development of reproductive tract neoplasms in domestic cats, while the reproductive management of threatened or endangered zoo-maintained felids requires safe and reversible contraception [23]. Indeed, contraception limits the reproduction of lesser genetically valuable felids and implements conservation objectives of species survival plans [23]. Therefore, as reproductive cancer hampers fertility and maintaining fertility in zoo felids is essential for captive breeding programs, it is important to regularly monitor animals for neoplasia development. Thorough physical exams and new non-invasive technologies as early detection will be the key to successful management of neoplasms which, based on published papers, most likely appear to be malignant in captive *Panthera* species.

## 4. Conclusions

Here, we have described a case of ovarian leiomyoma as an accidental finding during necropsy in a white tiger, detailing pathological and immunohistochemical findings and discussing the possible aetiology. Research into the role of widely used contraceptives to control reproduction in non-domestic felids is needed to shed light on risk factors for the development of reproductive system tumours in these animals, and health monitoring is warranted to support wildlife conservation. Investigations on wild and captive felids contribute to a better knowledge and understanding of the tumour types developed by these species, paving the way for early diagnosis and effective management.

## Data Availability

Not applicable.
